# **Elucidation of the bacterial communities associated with the harmful microalgae*Alexandrium tamarense*and*Cochlodinium polykrikoides*using nanopore sequencing**

**DOI:** 10.1038/s41598-018-23634-6

**Published:** 2018-03-28

**Authors:** HyeonSeok Shin, Eunju Lee, Jongoh Shin, So-Ra Ko, Hyung-Seok Oh, Chi-Yong Ahn, Hee-Mock Oh, Byung-Kwan Cho, Suhyung Cho

**Affiliations:** 1 Department of Biological Sciences, Korea advanced institute of Science and Technology, Daejeon, Republic of Korea; 2 KI for the BioCentury, Korea Advanced Institute of Science and Technology, Daejeon, Republic of Korea; 3 Biological Resource Center, Korea Research Institute of Bioscience and Biotechnology, Daejeon, Republic of Korea; 4 Intelligent Synthetic Biology Center, Daejeon, 34141 Republic of Korea

## Abstract

Interactions between microalgae and bacteria are often obligatory for harmful algal blooms (HABs). Here, we investigated the specific bacterial communities associated with *Alexandrium tamarense* and *Cochlodinium polykrikoides*, which cause ecological and economic damage during their blooms. To this end, the bacterial metagenome was selectively isolated from the two dinoflagellates and subsequently used for 16S rRNA analysis via the Nanopore MinION and Illumina sequencing platforms. Although the full-length 16S rRNA reads from the MinION platform showed high correlation in higher taxonomic ranks to the partial-length 16S rRNA reads from the Illumina platform, there was less correlation at the genus and species levels. MinION reads that are similar in the V3-V4 hypervariable regions with Illumina reads are classified to different taxonomies due to the extra information encoded in the full-length 16S rRNA reads. This indicates that bias arising from the short length Illumina reads can be supplemented by MinION reads. Furthermore, integrated analysis of the Illumina and MinION data showed that *A. tamarense* was predominantly enriched in the *Roseobacter* clade and *C. polykrikoides* was enriched in *Gammaproteobacteria* and *Alphaproteobacteria*. These results suggest that the association of different bacterial communities with *A. tamarense* and *C. polykrikoides* may be required for HABs.

## Introduction

Harmful algal blooms (HABs) are annually recurring phenomena that cause environmental and economic damage to marine environments and industries worldwide^1–4^. HABs are affected by multiple environmental factors, such as nutrients, temperature, light, water chemistry, and bacterial community^5–7^. Among those, bacterial communities associated with HABs were detected using high-throughput sequencing, catalysed reporter deposition-fluorescence *in situ* hybridization (CARD-FISH), and cell growth, which implies specific roles of the bacterial communities on HABs^8–11^. In particular, microalgal dependence on their associated bacterial community has been recognized as a unique mechanism of nutrient recycling and growth regulation^6,12–14^. Many studies have shown that microalgae depend on bacteria for the uptake of nutrients, such as nitrogen^12^, iron^15^, sulfur^16,17^, and vitamin B12^13^, indicating that their interaction is more complex and significant than expected^16,18^.

To better understand this interaction, 16S rRNA sequencing is often deployed to analyze bacterial diversity during algal blooms^1,19,20^. Although such analyses of environmental samples have provided valuable information on the diversity of free-living bacteria, it is difficult to identify the specific bacteria that interact with target algae^21^. The interaction of free-living bacteria with blooming algae is not obligatory and their composition often changes because of other environmental factors, such as temperature and nutrients^22^. Furthermore, analysis of the particle-attached bacteria is also unspecific in that diverse organisms in addition to blooming microalgae exist in the marine environment. Thus, identification of the specific interactions between target microalgae and their associated bacteria would provide valuable information.

Currently available methods to determine the bacterial diversity rely mostly on the Illumina-based sequencing of short, hypervariable regions in the 16S rRNA^23^. While the sequencing depth and accuracy of Illumina sequencing have great advantages over other sequencing methods, the length of the sequencing reads are limited^24^. The length limitation may lead to analytical bias, depending on the complexity of the bacterial community (i.e., sequence diversity of their hypervariable regions)^25^. Furthermore, differences in operation taxonomic unit (OTU) richness and distance levels have been observed, depending on the hypervariable regions^25^. To overcome this limitation, long-read sequencing platforms such as MinION and PacBio, which can obtain full-length 16S rRNA sequences, have been used^26–28^. Recently, 16S rRNA gene analysis of bacterial community have been investigated from ice wedge, blood, farm soil, and mouse gut using the nanopore technology^28–31^. The longer read allows detailed bacterial community characterization, down to the family or even genus level; however, the accuracy and sequencing output is limited compared to reads obtained using a shorter-read platform^26–28^. Thus, we used both shot- and long-read sequencing platforms to analyze the bacterial communities associated with *Alexandrium tamarense* and *Cochlodinium polykrikoides*, two toxin-producing dinoflagellates that affect human health by paralytic shellfish poisoning and kill fish through the production of reactive oxygen species, respectively^32,33^.

## Results and Discussion

### Metagenome isolation and sequencing

**Figure 1:**
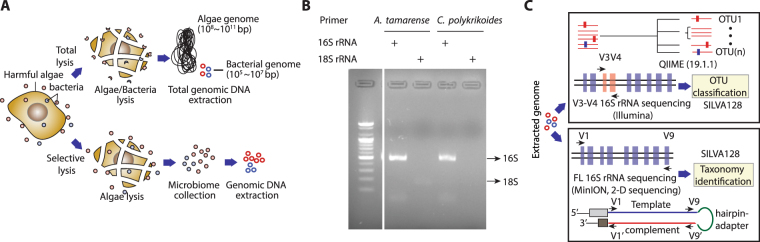
Metagenome isolation and sequencing library construction. (**A**) A schematic diagram of the bacterial metagenome isolation. The large dinoflagellate genome size and DNA content per cell is represented in the top and the enrichment of the bacterial metagenome by the lysis of microalgae is represented on the bottom. (**B**) PCR amplification results from the 16S rRNA and 18S rRNA gene amplification from the isolated bacterial metagenome. (**C**) Schematic diagram showing the different method characteristics and the analytic pipeline for 16S rRNA metagenome analysis using Illumina and MinION sequencing platforms.

To identify the bacteria associated with microalgae, we pre-cultivated *A. tamarense* and *C. polykrikoides* in laboratory conditions of sterile F/2 media, 100 μmolE m^−2^ s^−1^ light intensity, and 20 °C with an alternating 12 h light and dark cycle. Cultured cells were sampled at cell counts of 1,183 cells mL^−1^ and 1,100 cells mL^−1^ for *A. tamarense* and *C. polykrikoides*, respectively. The bacterial cells specifically associated with *A. tamarense* and *C. polykrikoides* were isolated by selective disruption of the eukaryotic microalgae, while the bacterial cells remained mostly intact (Fig. 1A). The clear enrichment of bacterial 16S rRNAs confirmed the depletion of the microalgal DNA and sequencing library construction of the purified bacterial DNA was performed (Figs 1B and S1). The isolated bacterial DNA was subjected to 16S rRNA sequencing using the Illumina and MinION platforms (Fig. 1C). For the Illumina platform, the sequencing library targeted the V3 and V4 hypervariable regions and sequencing was performed via MiSeq with 2 × 250 cycles for paired-end sequencing. After quality filtering, approximately 97% of the paired reads were successfully merged to a length of 447.4 bp (Table S1). Alternatively, the MinION platform offers a sufficiently long read length that spans the entire 16S rRNA gene. Thus, the MinION sequencing library was prepared using the primers S-D-bact-0008-c-S20 and S-D-bact-1391-a-A-17, which covers all of the hypervariable regions (V1 to V9)^28^. Sequencing of the four MinION libraries resulted in 177,691 high-quality passing reads from Metrichor, which are comprised of complementary reads merged to form more accurate 2D reads (Table S1). The 2D read generation from the raw data resulted in 68,870 2D reads with an average length of 1241.9 bp, which indicates an average 2.7× increase in length coverage. Sequencing the same metagenome with different PCR primers, library construction methods, and sequencing methods provides an additional frame of reference to cross-validate both the Illumina and MinION datasets. Furthermore, the quantitative ability of the sequencing methods was verified using an *in vitro* mock community analysis (Fig. S2).

### Taxonomy classification based on Illumina and MinION sequencing reads

The taxonomy classification of the Illumina reads by the QIIME pipeline resulted in 270 and 227 operational taxonomic units (OTUs) for *C. polykrikoides* and *A. tamarense*, respectively (Dataset S1)^34^. To determine if the number of identified OTUs were sufficient, we plotted a rarefaction curve using the inverse Simpson diversity estimation (Fig. S3). The bacterial diversity accumulation showed a steep increase up to 100 and 150 OTUs for *A. tamarense* and *C. polykrikoides*, respectively, indicating sufficient sequencing depth was acquired for all data. To validate the identified OTUs, we next investigated the ten most abundant species-dwelling locales. For both *A. tamarense* and *C. polykrikoides*, the identified habitats for the top ten species were in marine environments (Table S2). Among these species, *Roseovarius halotolerans*, *Marivita hallyeonensis*, *Ponticoccus litoralis*, and *Marinobacter flavimaris* were isolated from Korean coastal environments, indicating that this data robustly represent the experimental sample.

The MinION reads showed an average Phred quality score of 12.65 and the average median value for all samples was 10.26 (Fig. S4A–D). This result demonstrated that MinION reads are relatively inaccurate compared to the Illumina reads, which refrain from the use of existing metagenome analytic pipelines and require at least 97% identity. To this end, we used the LAST alignment program^35^ to directly aligned the MinION reads to the same SILVA 128 database that was used to analyze the Illumina reads (Fig. 1C). An average of 11,910 and 14,076.5 reads was successfully aligned to the reference database with at least 80% identity for *A. tamarense* and *C. polykrikoides*, respectively (Table S1)^26^. This resulted in the identification of 393 and 405 taxons for *A. tamarense* and *C. polykrikoides*, respectively (Dataset S2**)**.

### Comparative analysis of the MinION and Illumina data

**Figure 2:**
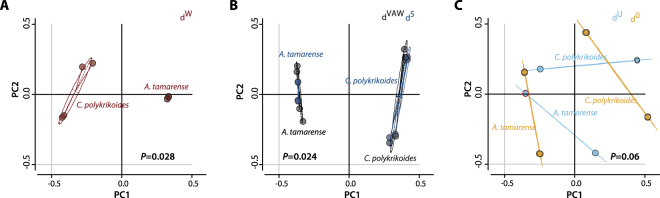
Comparison of the phylogenetical differences between samples. The generalized UniFrac PCoA plots grouped with the associated microalgae as the discriminating factor. The PCoA plots are based on (**A**) weighted UniFrac distance (d^W^), (**B**) moderately-weighted UniFrac distance (d^VAW^), and (**C**) unweighted UniFrac distance (d^U^). d^0.5^ and d^0^ indicate the generalized UniFrac distance of d^(α)^, where α controls the contribution of high-abundance branches ranging from 0 to 1. The perMANOVA *p*-value represents the statistical significance between microbial communities identified from *A. tamarense* and *C. polykrikoides*, regardless of the sequencing method.

To determine whether the bacterial communities identified by Illumina and MinION data correlate, we compared the relative abundance and phylogenetic distance between samples using the merged OTU table, where rare species <4 were removed (Dataset S3). The relative abundance of the bacterial community per taxonomic rank, from the class to the family level, showed a high correlation between MinION and Illumina data for both *A. tamarense* and *C. polykrikoides* (Fig. S5A,B). The Spearman’s rank correlation coefficient (*ρ*) values were greater than 0.36 and 0.71 for all taxonomic ranks higher or equal to the family level for *A. tamarense* and *C. polykrikoides*, respectively. However, at the genus and species levels, we found that the *ρ* values were reduced to less than 0.27, indicating differences in abundance at lower taxa levels. Consistent with the relative abundance comparison, the weighted and moderately weighted UniFrac distance plots show strong correlation for each sample grouped to their microalgae species. The *p* value from the perMANOVA analysis of the *A. tamarense* and *C. polykrikoides* were 0.028 and 0.02 for weighted UniFrac and moderately weighted UniFrac distance plots, respectively (Fig. 2A,B). This indicates that there was a significant difference between the samples grouped into *A. tamarense* and *C. polykrikoides*. Alternatively, the unweighted UniFrac distance (d^U^ and d°) plot showed a high perMANOVA *p* value of 0.060 (Fig. 2C). This result indicates that without factoring in the abundance, phylogenetic distance is biased and results depend on the sequencing or differences in the analytic pipeline, as the presence and absence of identified OTUs become the most sensitive factors.

### Bias analysis of the MinION and Illumina data

**Figure 3:**
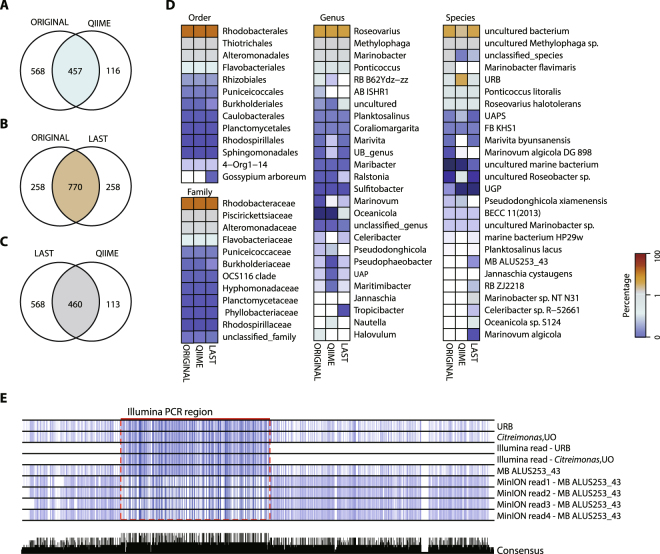
Comparison of the methodological differences between samples. Venn diagrams showing the number of differentially classified taxonomies after analyzing the recreated dataset for (**A**) ORIGINAL vs. QIIME, (**B**) ORGINAL vs. LAST, and (**C**) LAST vs. QIIME. ORIGINAL represents the recreated dataset of 1,028 error-free sequences obtained from the SILVA128 database of the bacterial taxonomy identified by the Illumina and MinION reads for *A. tamarense* and *C. polykrikoides*. LAST represents the results obtained by analyzing the ORIGINAL dataset with LAST alignment. QIIME represents the results obtained by analyzing the ORIGINAL dataset with the QIIME pipeline. (**D**) A heatmap of the relative abundance percentage change after analyzing the ORIGINAL data with the QIIME and LAST pipeline. The taxonomic ranks, from order to species, are shown for the taxonomies with an abundance level of at least 0.5%. (**E**) Example of sequence alignment composed of Illumina read and MinION reads for their representative sequences obtained from the SILVA128 database. (Top to Bottom) URB (EU328076.1.1447) sequence, *Citreimonas*, UO (EU246244.1.1424) sequence, Illumina reads classified as URB, Illumina reads classified as *Citreimonas*, UO, MB ALUS254_43 (E. AF359526.1.1401) sequence, MinION reads (1 and 2) classified as MB ALUS254_43 with a higher than 97% identity to URB sequence, and MinION reads (3 and 4) classified as MB ALUS254_43 with a higher than 97% identity to *Citreimonas*, UO sequence. The colors of the alignment represent the percentage identity between sequences. The consensus bar plot at the bottom represents the percentage of conserved sequences in the alignment. Abbreviations: AB ISHR1: *alphaproteobacterium* ISHR1; BECC 11(2013): bacterium enrichment culture clone 11(2013); FB KHS1: *Flavobacteriaceae* bacterium KHS1; MB ALUS253_43: marine bacterium ALUS253_43; RB MOLA13: *Rhodobacteraceae* bacterium MOLA 13; RB ZJ2218: *Rhodobacteraceae* bacterium ZJ2218; UAP: uncultured alphaproteobacterium; UAPS: uncultured alphaproteobacterium species; UB_genus: uncultured bacterium_genus; UGP: uncultured gamma proteobacterium; UO: uncultured organism; URB: uncultured *Rhodobacteraceae* bacterium.

The discrepancy at the lower taxa levels may be due to the short read length of the Illumina reads and the differences between the experimental and analytical pipelines. To test the potential bias caused by the analytical pipeline and the short sequencing length, we generated error-free sequences of the identified bacterial community from *A. tamarense* and *C. polykrikoides* and extracted only the Illumina sequencing target region that spans the V3-V4 hypervariable region to analyze the samples by LAST alignment and the QIIME pipeline. Among the 1,028 sequences recreated from the database, we found that 770 sequences were classified into the original taxonomy IDs. However, 258 taxonomies were classified differently by LAST alignment (Fig. 3A). This result indicates that at least 258 taxonomies had an identical sequence in the V3-V4 hypervariable region and approximately 25.10% of the reads were classified into a different taxonomy group due to the length limitation. The recreated analysis by QIIME showed an even more drastic difference when comparing to the original taxonomy (Fig. 3B). Due to the clustering step with 97% identity, 1,028 unique sequences were reduced to 573 sequences, among which 116 sequences were classified into a different taxonomy group. The taxonomy comparison obtained from LAST alignments and the QIIME pipeline showed similar results to the original QIIME taxonomy comparison results (Fig. 3C**)**. We next investigated the changes made in the abundance at different taxonomic ranks by generating a percentage heatmap (Fig. 3D). At higher taxonomic ranks, the changes in the predominant groups are not visible, which indicates that the differentially classified taxonomies are phylogenetically closely related. However, in the low abundance taxonomies, data regenerated from QIIME pipeline showed clustering of sequentially similar taxonomies into a single taxonomy, which led to the overestimation of uncultured *Rhodobacteraceae* bacterium species (Fig. 3D).

We also investigated whether PCR-based amplification led to experimental bias. Neither the number of mismatches between the primers and the sequence from the identified taxonomy nor the GC content of the identified taxonomy showed correlation with taxonomy abundance (Fig. S6A–D). These data indicate that the observed redundancy in the V3-V4 hypervariable region may lead to inaccurate taxonomic classification and that the analytical method for MinION data, which uses LAST alignment, shows relatively less bias when classifying organisms into lower taxonomic ranks.

In addition to the redundancy of the V3 and V4 hypervariable regions, sequences with similarity greater than 97% may lead to miscorrelation between the Illumina and MinION reads at the species level (Fig. S5A,B). Accordingly, we next investigated the similarity between MinION reads and Illumina reads using BLAST to compare the Illumina representative sequences against the MinION reads. Among Illumina reads with at least a 97% identity to MinION reads, 33 taxonomies were uniquely found only from the Illumina data. This difference can be occurred by the sequence discrepancy in the other hypervariable regions excluding V3-V4 region, since the taxonomic classification based on the MinION reads is generated from V1-V9 hypervariable regions. For example, the Illumina representative sequence for the taxonomy IDs EU328076.1.1447 (*Roseovarius*, uncultured *Rhodobacteraceae* bacterium) and EU246244.1.1424 (*Citreimonas*, uncultured organism) show a higher than 97% identity to 79 MinION reads that were classified to AF359526.1.1401 (*Roseovarius*, marine bacterium ALUS253_43) (Fig. 3E). The alignment of these reads, along with their reference sequences, shows that the OTUs EU328076.1.1447, EU246244.1.1424, and AF359526.1.1401 are indeed, very similar. These data indicate that the full-length MinION reads are advantageous at classification at the species level. It is however, difficult to conclude that a species identified from the MinION reads is the absolute correct species as the 16S rRNA database is not complete. Lack of an organism’s sequence in the database will lead to the classification of another species that has the most similar sequence.

### Bacterial community associated with *A. tamarense* and *C. polykrikoides*

**Figure 4:**
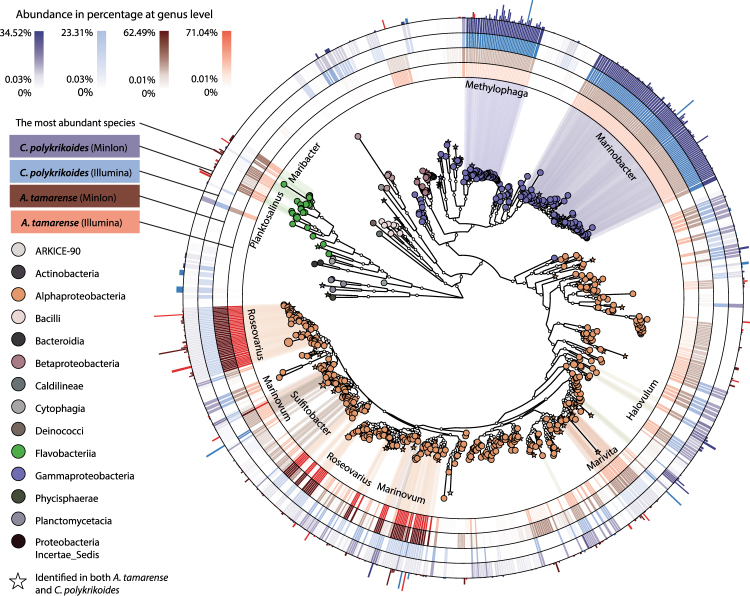
A phylogenic map of the microbial community associated with *A. tamarense* and *C. polykrikoides*. The clade colors represent the taxonomic identification at class level and the clade size represents the relative abundance for the combined library. The clade with the top ten abundant genus are linked from the clade to the inner ring where the relative abundance at the genus level is shown. The four rings indicate the abundance of the clade at the genus level for *C. polykrikoides* (MinION), *C. polykrikoides* (Illumina), *A. tamarense* (MinION), and *A. tamarense* (Illumina) in the order of outer to inner ring. The most outer ring bar length shows the relative abundance of the species that has the maximum abundance for each clade and the bar color represents the most abundant library.

*A. tamarense and C. polykrikoides*, each shows a preference for bacterial community composition. The bacterial community associated to *A. tamarense* showed a very strong enrichment in the *Roseovarious*, *Marinovum*, *Marivita*, and *Ponticoccus* genus, which all belong to the *Roseobacter* clade (Fig. 4)^16^. In total, 86% of the total bacterial population associated with *A. tamarense* was from this clade. This represents a steep increase in abundance compared to several reports that found the basal level of *Roseobacter* was 27–35% in environmental samples^17,36^. The steep increase in abundance of the *Roseobacter* clade in laboratory cultured *A. tamarense* grown to a very high concentration indicates that there is a mutualistic relationship between the *Roseobacter* clade and *A. tamarense* growth. Although the *Roseobacter* clade is known to be a universal player involved in key biogeochemistry processes, such as the recycling of several nutrients, including carbon, nitrogen, and phosphorus, the *Roseobacter* clade’s most dedicated role involves the recycling of sulfur^37^. From this result, we can deduce that one of the growth promoting mechanisms of *A. tamarense* may involve sufficient uptake of transformed sulfur, such as sulfonate^38^. Within the *Roseobacter* clade, the *Roseovarius* genus comprises more than 67% of the total bacterial community. In accordance with the *Roseobacter* general characteristics of sulfur transformation, the *Roseovarius* genus is known to contain the Sox multienzyme complex in the periplasmic region. The oxidation of thiosulfate, which is the most abundant form of reduced sulfur in seawater, is hypothesized to be one of the main sources of energy for the bacteria, recycling the transformed sulfur into the environment. Furthermore, the interaction between the *Roseovarius* genus and microalgae via attachments and quorum sensing is also one of the characteristics of the *Roseobacter* clade^39^.

*Flavobacteria*, bacteria that belong to the *Flovabacteriia* class, were the second most abundant group that was associated with *A. tamarense*. The *Roseobacter* and *Flavobacteria* groups comprise 95% of the total bacteria found in *A. tamarense*. These data agree with the previous notion that the abundance of *Roseobacter* and *Flavobacteria* with algal blooms correlate, which suggests that their relationship is mutualistic and potentially obligate^21^.

On the other hand, *Gammaproteobacteria* and *Alphaproteobacteria* were the main composition of the bacterial community associated with *C. polykrikoides*, comprising 53% and 40% of the total population, respectively (Fig. 4). Compared to the findings from a previous study that the basal level of *Gammaproteobacteria* and *Alphaproteobacteria* from the environment amounted to 37.3% and 31.4% respectively, our findings indicate an enrichment of *Gammaproteobacteria* and *Alphaproteobacteria* with a corresponding diminution of flavobacteria^40^. Compared to the *A. tamarense-*associated bacterial composition, *C. polykrikoides* interact with a more diverse bacterial population, especially at the lower taxa levels. While the *Roseovarius* genus was almost exclusively enriched in *A. tamarense*, the four most abundant genera associated with *C. polykrikoides* include *Methylophaga*, *Marinobacter*, *Ponticoccus*, and *Jannaschia*, which comprise 62% of the total bacterial community. This data indicate that *C. polykrikoides*-associated bacteria are more diverse and complex than those associated with *A. tamarense*. Additionally, the stronger enrichment of *C. polykrikoides* to *Gammaproteobacteria* suggests that the mechanistic interaction between the microalgae and their associated bacteria is clearly different. For example, the enriched *Marinobacter* genus in *C. polykrikoides* cultures contains metabolic facilities to transform iron in the form of Fe^3+^ and a symbiotic vitamin B12 transporter, which hints to a symbiotic relationship with the *Marinobacter* genus^14,41–44^.

Overall, this study uses both the Illumina and MinION platforms to identify the bacterial communities associated with *A. tamarense* and *C. polykrikoides*, the two microalgae that frequently cause HABs around the globe. The combined Illumina and MinION data identified 437 and 386 OTUs associated with *A. tamarense* and *C. polykrikoides*, respectively. While the variations in taxonomy classifications at the lower taxa level were observed, we demonstrated that this variance was not caused by the experimental methods or the MinION data analytical pipeline. Evaluation of the sequencing methods and their analytical pipelines showed that the full-length MinION reads allow a more accurate estimation of the abundance of each bacterial species. The *A. tamarense*-associated bacterial community was enriched in the *Roseobacter* clade, while the *C. polykrikoides* bacterial community was enriched in *Gammaproteobacteria* and *Alphaproteobacteria*, suggesting different bacterial associations.

## Methods

### Cell culture and metagenome isolation

*C. polykrikoides* and *A. tamarense* were collected from surface water near Tongyeong and Namhae of South Korea, respectively. A single *C*. *polykrikoides* and *A*. *tamarense* was isolated using a micropipetting method and then maintained in F/2 media at 20 °C with a 12 h light/dark cycle using a light intensity of 100 μmolE m^−2^ s^−1^, subculturing with a 1/10 dilution approximately 24 and 36 times, respectively. Cell counts were measured on microscope slide-grids. The cultures were harvested for the metagenome experiment when *C. polykrikoides* and *A. tamarense* reached 1,100 cells mL^−1^ and 1,183 cells mL^−1^, respectively. Cells were collected on 0.45 µm Whatman^®^ membranes by filtration. In order to specifically lyse the eukaryotic microalgae, the collected cells were lysed using the lysis buffer in the QIAamp DNA microbiome kit (Qiagen), followed by centrifugation at 10,000 × g for 10 min. The bacterial metagenome was then purified from the lysate using the QIAamp^®^ DNA microbiome kit, according to the manufacturer’s instructions.

### Illumina sequencing library construction

The 16S rRNA sequencing libraries for Illumina and MinION sequencing were constructed as previously described^28^. The protocol for 16S metagenomic sequencing library preparation (Illumina, San Diego, CA, USA) was used to construct the sequencing library for the V3 and V4 hypervariable regions of the 16S rRNA gene. Briefly, the isolated metagenomic DNA was used as a template to amplify the V3 and V4 hypervariable regions of the 16S rRNA gene using the following set of primers: 5′-TCGTCGGCAGCGTCAGATGTGTATAAGAGACAGCCTACGGGNGGCWGCAG-3′ and 5′-GTCTCGTGGGCTCGGAGATGTGTATAAGAGACAGGACTACHVGGGTATCTAATCC-3′. The amplified DNA was purified by AMPure XP magnetic beads and subsequently amplified using the Nextera XT Index kit (Illumina) for sequencing adaptor integration. The generated library was quantified with the Qubit dsDNA HS Assay Kit (Thermo Fisher Scientific) using a Qubit 3.0 fluorometer (Invitrogen) and DNA was pooled to a concentration 2 nM. The pooled library was denatured with 0.2 N NaOH, diluted further to 5 pM, combined with 15% (v/v) denatured 5.2 pM PhiX, and sequenced on the MiSeq sequencing platform with a 2 × 250 cycle V2 kit.

### MinION sequencing library construction

To construct the 16S rRNA metagenome sequencing library for the MinION, the isolated metagenomic DNA was amplified using the S-D-bact-0008-c-S20 (5′-AGRGTTYGATYMTGGCTCAG-3′) and S-D-bact-1391-a-A-17 (5′-GACGGGCGGTGWGTRCA-3′) primers for broad range amplification of the 16S rRNA gene. The 16S rRNA amplification was monitored using CFX96 Real-Time PCR (Bio-Rad, Hercules, CA, USA) up to the saturation point (2 times) and the amplified DNA was purified using the MinElute Gel Extraction Kit (Qiagen, Venlo, Netherlands) (Fig. S1B). The purified amplicons were end-repaired using the NEBNext^®^ End Repair Module (NEB, Ipswich, MA, USA), and subsequently dA-tailed using the NEBNext^®^ dA-Tailing Module (NEB), based on the SQK-LSK208 protocol (Oxford Nanopore Technologies, Oxford, UK). For MinION sequencing, the dA-tailed amplicon adapters were ligated using the provided adapter mix from Oxford Nanopore Technologies and the DNA was purified. The sequencing data were deposited on the European Nucleotide Archive with accession code: PRJEB22027.

### Illumina data analysis

To analyze the microbial community from the Illumina sequencing data, the script from QIIME (version 1.9.1) was used^34^. The paired-end reads were joined using the multiple_join_paired_ends.py script and quality filtered by the multiple_split_libraries_fastq.py script. The OTUs were clustered using UCLUST, with a default threshold of 0.97. The OTU phylotype was assigned using the closed-reference OTU picking method against the QIIME modified version of the SILVA 128 database.

### MinION data analysis

Base calling of the MinION data was performed using the FLO-MIN106 250 bps workflow, which is a 2D base calling method. The base-called, high quality, passed reads were then screened to obtain the 2D reads using poretools^45^. The screened 2D reads were aligned against the QIIME modified SILVA 128 database using LAST aligner with the following scoring parameters: match score +1, gap opening penalty −1, and gap extension −1. The generated mutation annotation format (MAF) files were reformatted to axt alignment format. In-house scripts were used for further analysis of bacterial abundance.

### Integrated analysis of Illumina and MinION data

The combined analysis of Illumina and MinION data was performed based on the merged table of Illumina and MinION data after removal of rare OTUs, where OTUs with low abundance <4 were removed (Dataset S3). The uniquely identified taxonomy sequences were aligned to generate a phylogenic tree using the FastTree method and the midpoint rooted method with the QIIME script make_phylgeny.py. GUniFrac was used to measure the UniFrac distance based on the generated phylogenic tree^46^. The heatmaps were separately generated using the relative percentage abundance for each taxonomic rank, comparing Illumina and MinION data. To test if the Illumina and MinION libraries contain any bias from the different PCR primers, virtual PCR of the database with a different number of mismatches was performed using a script from MOTHUR software called pcr.seqs.^47^. The phylogenetic tree for visualization by GraPhlAn was generated with maximum likelihood distance^48,49^.

## Electronic supplementary material

Supplementary information

Dataset 1

Dataset 2

Dataset 3
