# Supplementary material for: Elucidation of the bacterial communities associated with the harmful microalgaeAlexandrium tamarenseandCochlodinium polykrikoidesusing nanopore sequencing
Source: Sci Rep. 2018 Mar 28;8:5323. doi: 10.1038/s41598-018-23634-6 (PMC5871755; doi:10.1038/s41598-018-23634-6)
Supplement: Supplementary file 1 — Supplementary information [file 41598_2018_23634_MOESM1_ESM.pdf]

Supplementary Information

Elucidation of the bacterial communities associated with the harmful microalgae *Alexandrium tamarense* and *Cochlodinium polykrikoides* using nanopore sequencing

HyeonSeok Shin,[a,b] Eunju Lee,[a,b], Jongoh Shin [a,b], So-Ra Ko,[c] Hyung-Seok Oh,[c] Chi-Yong Ahn,[c] Hee Mock Oh,[c] Byung-Kwan Cho,[a,b,d*] and Suhyung Cho[a,b*]

Department of Biological Sciences, Korea advanced institute of Science and Technology, Daejeon, Republic of Korea[a]; KI for the BioCentury, Korea Advanced Institute of Science and Technology, Daejeon, Republic of Korea[b]; Biological Resource Center, Korea Research Institute of Bioscience and Biotechnology, Daejeon, Republic of Korea[c]; Intelligent Synthetic Biology Center, Daejeon 34141, Republic of Korea[d]

*Address correspondence to Suhyung Cho (shcho95@kaist.ac.kr) and Byung-Kwan Cho (bcho@kaist.ac.kr)

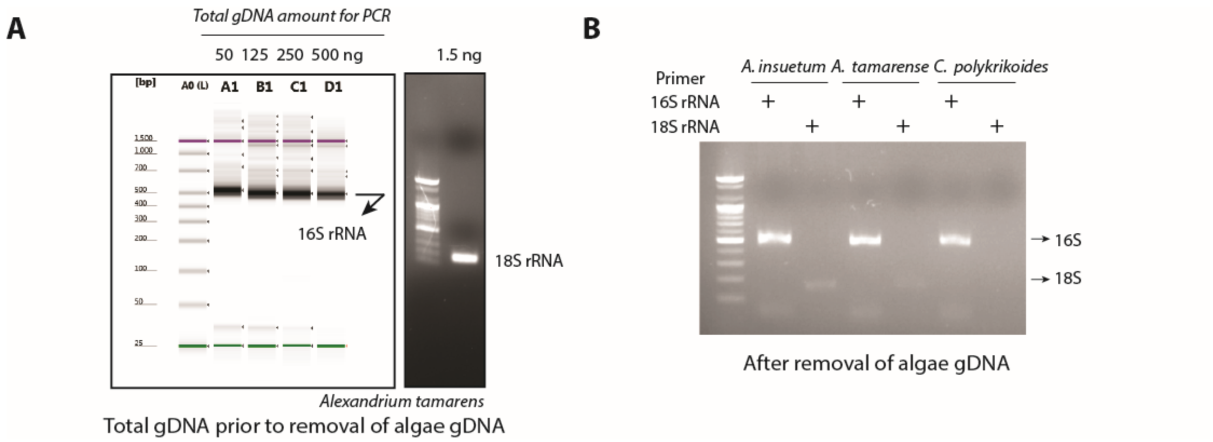

Supplementary Figure S1. The purification of bacterial genomic DNA from algae. A. After extraction of total genomic DNA using liquid nitrogen method from *Alexandrium tamarens*, the presences of 16S rDNA and 18S rDNA were confirmed using PCR. B. After selective removal of algae genomic DNAs from *A. insuetum*, *A. tamarense, and C. polykrikoides*, the presences of 16S rDNA and 18S rDNA were examined using 2% agarose gel electrophoresis.

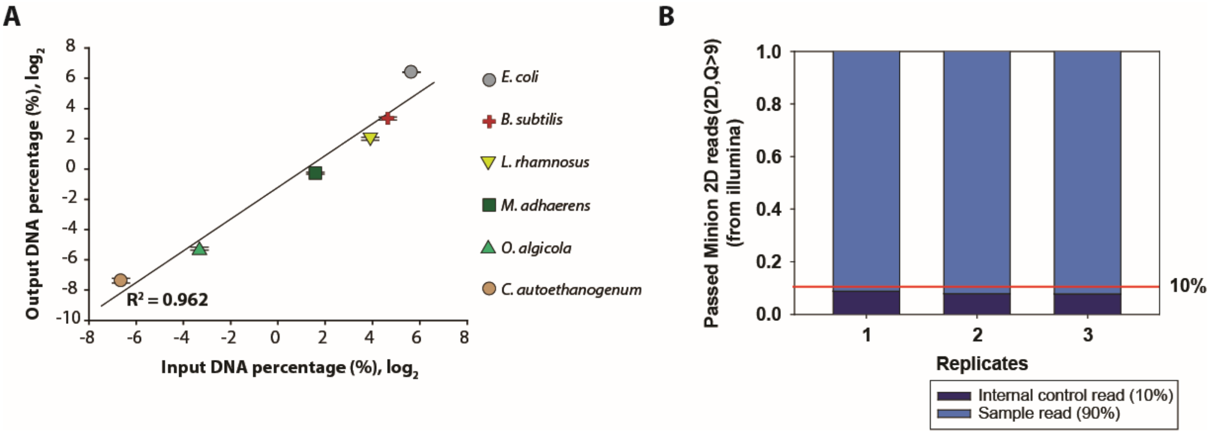

Supplementary Figure S2. Validation for two different sequencing methods, Illumina and MinION. A. Scatter plot of the abundance correlation between the percentage of input gDNA and the output gDNA amount matched to the database. For this result, *in vitro* microbial mock community was constructed using the gDNA extracted from the four general bacterial strains stored in the laboratory and two strains isolated from the marine coast. Sequences of all species were confirmed via Sanger sequencing prior to the experiment. The gDNA of each species were quantified by Qubit dsDNA broad range assay kit using Qubit 3.0 fluorometer (Invitrogen) and diluted to 2.5 ng ul^{-1} to make a mixture in the ratio of 50:25:15:3:0.1:0.01 with the following order: *Escherichia coli* K-12 MG1655: *Bacillus subtillis* KCTC strain 168: *Lactobacillus rhamnosus*: *Marinobacter adhaerens: Olleya algicola* KCTC22024: *Clostridium autoethanogenum*. The same protocol used in the manuscript for 16S metagenomic sequencing library preparation (Illumina) was used to construct the sequencing libraries for the V3 and V4 hypervariable regions in triplicate. Analysis of the sequencing results show that the abundance of the input DNA and the sequencing output correlate. B. The percentage conservativeness after the addition of 10% lamda DNA to mixed 16S rRNA microbiome amplicon was verified through the ratio of mapping reads of lamda DNA to total reads from MinION sequencing method.

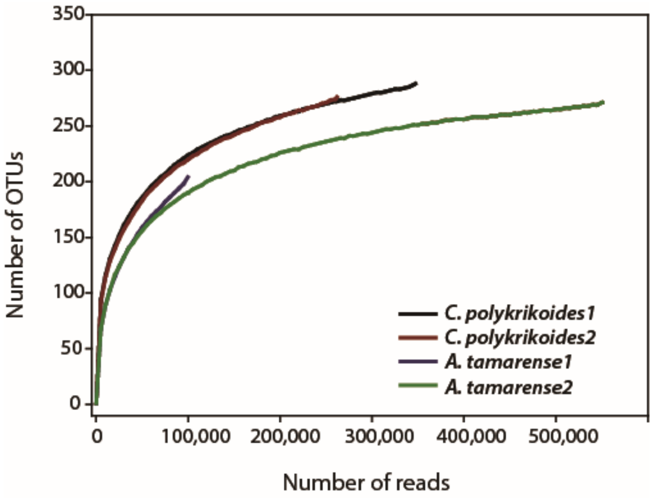

Supplementary Figure S3. The rarefaction plot of the Illumina reads with Simpson diversity estimation.

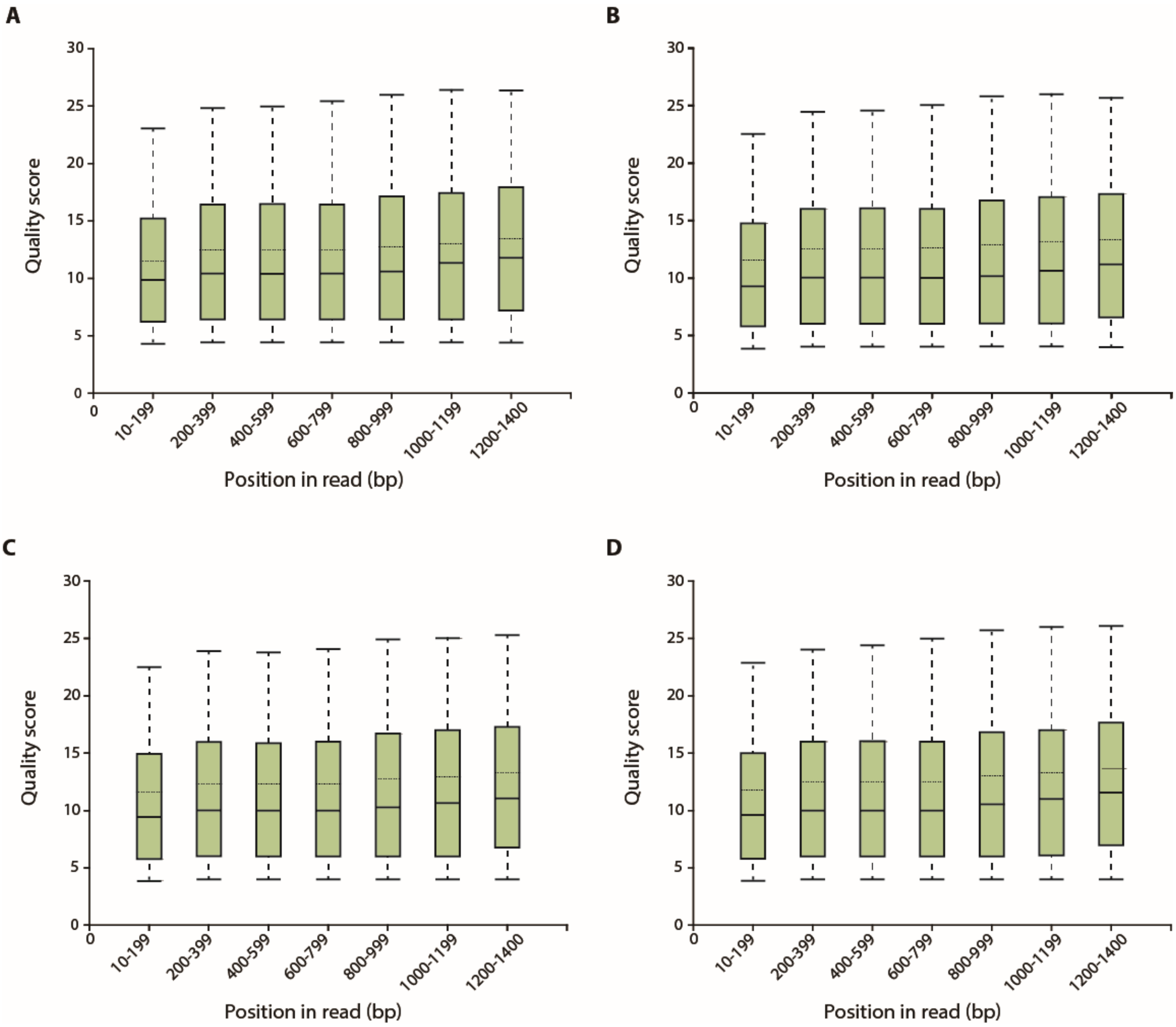

Supplementary Figure S4. The base quality of MinION sequencing results for (A) *C. polykrikoides* replicate 1, (B) *C. polykrikoides* replicate 2, (C) *A. tamarense* replicate 1 and (D) *A. tamarense* replicate 2. The 10th and 90th percentiles are marked by the error range and the median and mean values are shown by solid and dashed line inside the box plot, respectively. (E) The distribution of the alignment score of MinION reads aligned to the QIIME pipeline modified SILVA128 database.

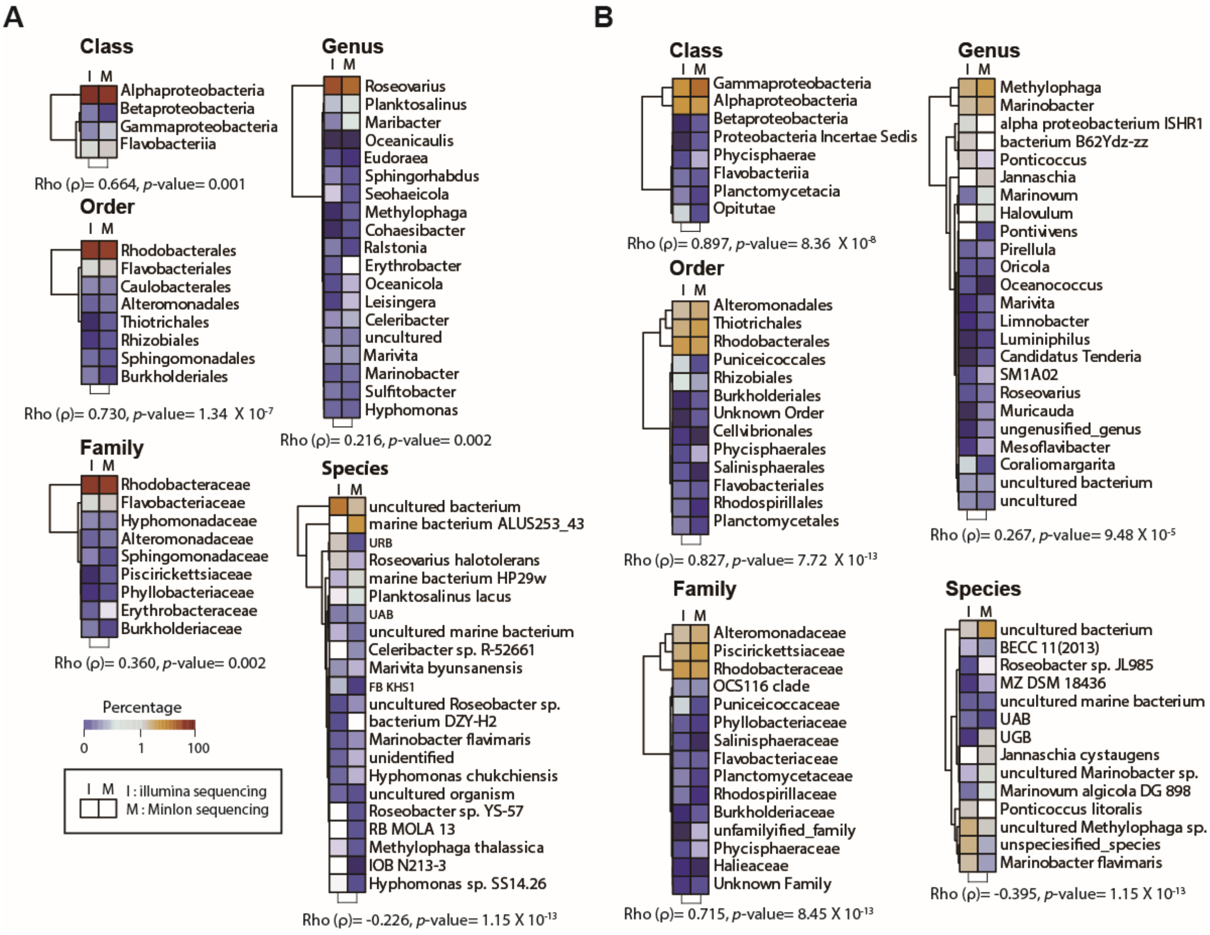

Supplementary Figure S5. A heatmap of the mean abundance value of (A) *A. tamarense* and (B) *C. polykrikoides* at taxonomic ranks of class, order, family, genus and species. The relative abundance is shown in percentage value. Only the taxonomies with an abundance level higher than 0.5% are shown. The ρ value indicates the Spearman Rank-Order correlation and the *P*-values indicate the validity of the correlation coefficients. For all comparisons, the *P*-values for Spearman Rank-Order correlation tests were less than 0.01. Abbreviations: BECC 11(2013): bacterium enrichment culture clone 11(2013); FB KHS1: *Flavobacteriaceae* bacterium KHS1;IOB N213-3:iodide-oxidizing bacterium N213-3; MZ DSM 18346: *Mesoflavibacter zeaxanthinifaciens* DSM 18436; RB MOLA13: *Rhodobacteraceae bacterium* MOLA 13; RB B62Ydz-zz: *Rhodobacteraceae bacterium* B62Ydz–zz; UAB: uncultured *Alphaproteobacterium*; UGB: uncultured *Gammaproteobacterium*; URB: uncultured *Rhodobacteraceae bacterium*.

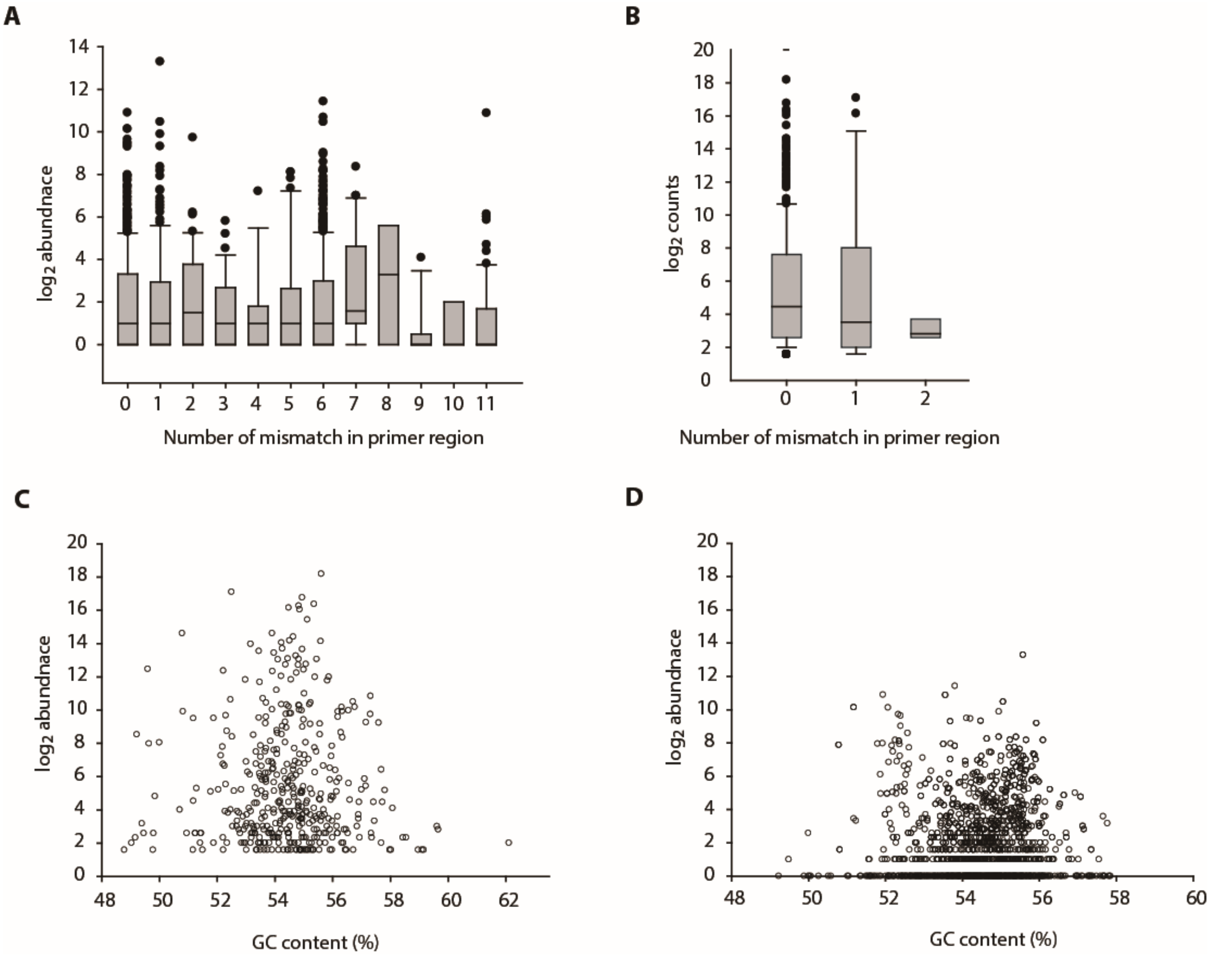

Supplementary Figure S6. Examination of PCR bias occurring from primer mismatch and GC contents. The boxplot shows the number of mismatches between the primers and the identified taxon's sequence for primers used for (a) MinION library and (b) Illumina library. The GC contents of the identified taxonomies and their abundance is plotted for (c) Illumina and (d) MinION library.

Supplementary Table S1. Illumina and MinION sequencing results of *A. tamarense* and *C. polykrikoides*

	Illumina reads statistics				MinION reads statstics			
	A. tamarense		*C. polykrikoides*		*A. tamarense*		*C. polykrikoides*	
Samples	# 1	# 2	# 1	# 2	# 1	# 2	# 1	# 2
Total number of raw reads	104,704	586,287	434,901	325,149	37,950	68,310	76,962	34,176
Average length of the raw reads (bp)	250.96	250.96	250.91	250.92	1,176.25	1,154.93	1,185.08	1,178.38
Total number of processed reads*	102,249	566,931	419,645	313,225	12,088	21,801	24,250	10,731
Average length of the processed reads* (bp)	442.38	442.65	451.49	453.19	1,237.46	1,217.89	1,259.36	1,252.76

* The processed reads refers to the quality filtered reads merged from the paired-ends reads to single contigs for Illumina reads and the template and complementary reads merged to 2D reads for MinION reads.

Supplementary Table S2. The top ten species identified from Illumina reads

OTU ID	Lowest identified taxonomy	Habitat
**Bacteria OTU abundant *in A. tamarense***		
EF632662.1.1477	Genus (*Roseovarius*)	Marine[1]
EU328076.1.1447	Genus (*Roseovarius*)	Marine[1]
EU431217.1.1430	Species (*Roseovarius halotolerans*)	Marine[2]
JF946744.1.1394	Species (*Flavobacteriaceae* bacterium KHS1)	Marine[3]
FJ467624.1.1422	Species (*Marivita byunsanensis*)	Marine[4]
KF268904.1.1396	Genus (*Maribacter*)	Marine[5]
JX095815.1.1394	Genus (*Ralstonia*)	Marine[6]
FJ202246.1.1405	Family (*Rhodobacteraceae*)	Marine[7]
EF471656.1.1422	Family (*Rhodobacteraceae*)	Marine[7]
AWFG01000025.396.1855	Species (*Hyphomonas chukchiensis*)	Marine[8]
**Bacteria OTU abundant in *C. polykrikoides***		
JX526708.1.1438	Genus (*Methylophaga*)	Marine[9]
EU440995.1.1506	Species (*Marinobacter flavimaris*)	Marine[10]
EF211829.1.1357	Species (*Ponticoccus litoralis*)	Marine[11]
EU070404.1.1347	Family (*Rhodobacteraceae*)	Marine[7]
AB013442.1.1373	Family (*Rhodobacteraceae*)	Marine[7]
FJ202515.1.1497	Genus (*Coraliomargarita*)	Marine[12]
KF771532.1.1451	Family (OCS116 clade)	Marine[13]
CP010855.220385.221727	Species (*Marinovum algicola* DG 898)	Marine[14]
JF272183.1.1489	Genus (*Pirellula*)	Marine[15]
EF092174.1.1291	Family (*Rhodospirillaceae*)	Marine[16]

Supplementary References

1 Choi, E. J., Lee, H. J., Kim, J. M. & Jeon, C. O. *Roseovarius lutimaris* sp. nov., isolated from a marine tidal flat. *Int. J. Syst. Evol. Microbiol.* **63**, 3835-3840 (2013).

2 Oh, Y. S. *et al. Roseovarius halotolerans* sp. nov., isolated from deep seawater. *Int. J. Syst. Evol. Microbiol.* **59**, 2718-2723 (2009).

3 Raymond, J. A., Christner, B. C. & Schuster, S. C. A bacterial ice-binding protein from the Vostok ice core. *Extremophiles* **12**, 713-717 (2008).

4 Yoon, J. H. *et al. Marivita hallyeonensis* sp. nov., isolated from seawater, reclassification of *Gaetbulicola byunsanensis* as *Marivita byunsanensis* comb. nov. and emended description of the genus *Marivita* Hwang et al. 2009. *Int. J. Syst. Evol. Microbiol.* **62**, 839-843 (2012).

5 Nedashkovskaya, O. I. *et al. Maribacter* gen. nov., a new member of the family *Flavobacteriaceae*, isolated from marine habitats, containing the species *Maribacter sedimenticola* sp. nov., *Maribacter aquivivus* sp. nov., *Maribacter orientalis* sp. nov. and Maribacter ulvicola sp. nov. *Int. J. Syst. Evol. Microbiol.* **54**, 1017-1023 (2004).

6 T, D. A. *et al.* The coral core microbiome identifies rare bacterial taxa as ubiquitous endosymbionts. *ISME J.* **9**, 2261-2274 (2015).

7 Simon, M. *et al.* Phylogenomics of *Rhodobacteraceae* reveals evolutionary adaptation to marine and non-marine habitats. *ISME J* **11**, 1483-1499 (2017).

8 Li, C. *et al. Hyphomonas beringensis* sp. nov. and *Hyphomonas chukchiensis* sp. nov., isolated from surface seawater of the Bering Sea and Chukchi Sea. *Antonie Leeuwenhoek* **106**, 657-665 (2014).

9 Schafer, H. Isolation of *Methylophaga* spp. from marine dimethylsulfide-degrading enrichment cultures and identification of polypeptides induced during growth on dimethylsulfide. *Appl.Environ. Microbiol.* **73**, 2580-2591 (2007).

10 Yoon, J. H., Yeo, S. H., Kim, I. G. & Oh, T. K. *Marinobacter flavimaris* sp. nov. and Marinobacter daepoensis sp. nov., slightly halophilic organisms isolated from sea water of the Yellow Sea in Korea. *Int. J. Syst. Evol. Microbiol.* **54**, 1799-1803 (2004).

11 Hwang, C. Y. & Cho, B. C. *Ponticoccus litoralis* gen. nov., sp. nov., a marine bacterium in the family Rhodobacteraceae. *Int. J. Syst. Evol. Microbiol.* **58**, 1332-1338 (2008).

12 Yoon, J. *et al. Coraliomargarita akajimensis* gen. nov., sp. nov., a novel member of the phylum 'Verrucomicrobia' isolated from seawater in Japan. *Int. J. Syst. Evol. Microbiol.* **57**, 959-963 (2007).

13 Treusch, A. H. *et al.* Seasonality and vertical structure of microbial communities in an ocean gyre. *ISME J.* **3**, 1148-1163 (2009).

14 Pradella, S., Pauker, O. & Petersen, J. Genome organisation of the marine Roseobacter clade member *Marinovum algicola*. *Arch. Microbiol.* **192**, 115-126 (2010).

15 Rabus, R. *et al.* Analysis of N-acetylglucosamine metabolism in the marine bacterium *Pirellula* sp. strain 1 by a proteomic approach. *Proteomics* **2**, 649-655 (2002).

16 Jiang, Z. Y., Rushing, B. G., Bai, Y., Gest, H. & Bauer, C. E. Isolation of *Rhodospirillum centenum* mutants defective in phototactic colony motility by transposon mutagenesis. *J. Bacteriol.* **180**, 1248-1255 (1998).
